# Intestinal autotransplantation for complex intra-abdominal diseases previously considered unresectable: a systematic review and single-arm meta-analysis

**DOI:** 10.1093/gastro/goag074

**Published:** 2026-07-17

**Authors:** Yierfan Yilihaer, Talaiti Tuergan, Bingwei Liu, Alimu Tulahong, Ainiwar Aikebair, Hao Wen, Tie-Min Jiang, Ying-Mei Shao, Rexiati Ruze, Tuerganaili Aji

**Affiliations:** Department of Hepatobiliary and Echinococcosis Surgery, Digestive and Vascular Surgery Center, First Affiliated Hospital of Xinjiang Medical University, Urumqi, Xinjiang 830054, P. R. China; State Key Laboratory of Pathogenesis, Prevention and Treatment of High Incidence Diseases in Central Asia, Xinjiang Medical University, Urumqi, Xinjiang 830054, P. R. China; Department of Hepatobiliary and Echinococcosis Surgery, Digestive and Vascular Surgery Center, First Affiliated Hospital of Xinjiang Medical University, Urumqi, Xinjiang 830054, P. R. China; State Key Laboratory of Pathogenesis, Prevention and Treatment of High Incidence Diseases in Central Asia, Xinjiang Medical University, Urumqi, Xinjiang 830054, P. R. China; Department of Hepatobiliary and Echinococcosis Surgery, Digestive and Vascular Surgery Center, First Affiliated Hospital of Xinjiang Medical University, Urumqi, Xinjiang 830054, P. R. China; State Key Laboratory of Pathogenesis, Prevention and Treatment of High Incidence Diseases in Central Asia, Xinjiang Medical University, Urumqi, Xinjiang 830054, P. R. China; Department of Hepatobiliary and Echinococcosis Surgery, Digestive and Vascular Surgery Center, First Affiliated Hospital of Xinjiang Medical University, Urumqi, Xinjiang 830054, P. R. China; State Key Laboratory of Pathogenesis, Prevention and Treatment of High Incidence Diseases in Central Asia, Xinjiang Medical University, Urumqi, Xinjiang 830054, P. R. China; Department of Hepatobiliary and Echinococcosis Surgery, Digestive and Vascular Surgery Center, First Affiliated Hospital of Xinjiang Medical University, Urumqi, Xinjiang 830054, P. R. China; State Key Laboratory of Pathogenesis, Prevention and Treatment of High Incidence Diseases in Central Asia, Xinjiang Medical University, Urumqi, Xinjiang 830054, P. R. China; State Key Laboratory of Pathogenesis, Prevention and Treatment of High Incidence Diseases in Central Asia, Xinjiang Medical University, Urumqi, Xinjiang 830054, P. R. China; Department of Hepatobiliary and Echinococcosis Surgery, Digestive and Vascular Surgery Center, First Affiliated Hospital of Xinjiang Medical University, Urumqi, Xinjiang 830054, P. R. China; State Key Laboratory of Pathogenesis, Prevention and Treatment of High Incidence Diseases in Central Asia, Xinjiang Medical University, Urumqi, Xinjiang 830054, P. R. China; Department of Hepatobiliary and Echinococcosis Surgery, Digestive and Vascular Surgery Center, First Affiliated Hospital of Xinjiang Medical University, Urumqi, Xinjiang 830054, P. R. China; State Key Laboratory of Pathogenesis, Prevention and Treatment of High Incidence Diseases in Central Asia, Xinjiang Medical University, Urumqi, Xinjiang 830054, P. R. China; Department of Hepatobiliary and Echinococcosis Surgery, Digestive and Vascular Surgery Center, First Affiliated Hospital of Xinjiang Medical University, Urumqi, Xinjiang 830054, P. R. China; State Key Laboratory of Pathogenesis, Prevention and Treatment of High Incidence Diseases in Central Asia, Xinjiang Medical University, Urumqi, Xinjiang 830054, P. R. China; Department of Hepatobiliary and Echinococcosis Surgery, Digestive and Vascular Surgery Center, First Affiliated Hospital of Xinjiang Medical University, Urumqi, Xinjiang 830054, P. R. China; State Key Laboratory of Pathogenesis, Prevention and Treatment of High Incidence Diseases in Central Asia, Xinjiang Medical University, Urumqi, Xinjiang 830054, P. R. China

**Keywords:** intestinal autotransplantation, *ex vivo* resection, complex intra-abdominal malignancy, mesenteric vessel involvement, single-arm meta-analysis

## Abstract

**Background:**

Intestinal autotransplantation (IATx) facilitates radical resection for advanced intra-abdominal cancers involving major vessels, which were previously deemed unresectable. This study systematically reviewed and meta-analyzed its perioperative safety and mid-term survival outcomes.

**Methods:**

A search of databases was conducted up to December 2025, adhering to PRISMA guidelines. Studies reporting outcomes of IATx were included in the review. A total of 12 studies involving 160 patients were reviewed, with meta-analysis performed on six studies comprising 122 patients, using random-effects models. Primary outcomes included the 3-year overall survival (OS) rate, 90-day mortality, major complications (Clavien–Dindo ≥ III), and R0 resection rates. Secondary outcomes involved cold ischemia time, operative time, and length of hospital stay. Study quality was assessed by using the MINORS criteria.

**Results:**

The pooled 3-year OS rate was 57.9% (95% CI, 34.1%–75.7%; 4 studies; *I*^2^ = 36.8%, *P *= 0.040). Analysis of perioperative outcomes from six studies (122 patients) revealed a pooled 90-day mortality of 6.6% (95% CI, 3.1%–13.5%; *I*^2^ = 0%), a major complication rate (Clavien–Dindo ≥ III) of 27.9% (95% CI, 12.9%–50.3%; *I*^2^ = 71.8%), and an R0 resection rate of 92.3% (95% CI, 84.3%–96.4%; *I*^2^ = 0%). The pooled mean cold ischemia time was 124 minutes (95% CI, 75–173 minutes; 5 studies; *I*^2^ = 98.9%), the operative time was 10.4 h (95% CI, 9.1–11.7 h; 6 studies; *I*^2^ = 90.4%), and the postoperative length of stay averaged 22 days (95% CI, 19–25 days; 5 studies; *I*^2^ = 35%).

**Conclusions:**

IATx for unresectable complex intra-abdominal tumors demonstrates high R0 resection rates, low mortality, and encouraging 3-year OS outcomes. Given the procedural complexity, IATx should be performed exclusively at expert centers. Further long-term follow-up studies are necessary to validate these findings.

## Introduction

Surgical resection plays a pivotal role in achieving long-term survival and potential cure for patients with locally advanced intra-abdominal malignancies. Radical surgery may be indicated for carefully selected patients following a comprehensive evaluation by a multidisciplinary team (MDT), aimed at improving outcomes [1, 2]. Tumors located in the pancreatic head, mesentery, or retroperitoneum that invade the superior mesenteric artery (SMA) or its major branches often present complex challenges, rendering conventional *in situ* resection inadequate. This complexity heightens the risk of prolonged mesenteric ischemia and irreversible organ damage, leading these cases to be frequently classified as unresectable [3, 4].

Recent advancements in vascular surgery and organ preservation have facilitated the application of intestinal autotransplantation (IATx) for tumors involving critical mesenteric vessels. Typically, the procedure involves the simultaneous resection of the tumor and affected intestine, followed by *ex vivo* tumor excision and vascular management under cold preservation. This is succeeded by autologous intestinal reimplantation with vascular anastomoses and gastrointestinal reconstruction [5]. This approach enhances vascular control, allows for improved tumor dissection, and provides superior exposure, resulting in a bloodless operative field. It also enhances the likelihood of achieving margin-negative (R0) resections while aiming to preserve intestinal structure and function. Recently, reports from various centers have highlighted technical modifications, such as perfusion-assisted *in situ* approaches, which may reduce ischemia time and enhance perioperative safety [6, 7].

The foundation of IATx lies in the advancements of intestinal transplantation and organ preservation techniques derived from transplant medicine. Over the years, enhancements in perioperative care, immunologic management, and preservation methods have made intestinal transplantation feasible at select centers. Building on these principles, Tzakis *et al.* [8] introduced IATx in 2000, employing transplant techniques to facilitate radical resections of tumors involving major mesenteric vessels while preserving organ function.

Despite an increasing number of reports, the evidence base surrounding IATx remains limited. Most published studies are small, single-center, and retrospective in nature. Outcomes such as mortality, major complications, ischemia time, and operative duration exhibit considerable variability attributable to differences in surgical strategies, including *ex vivo* autotransplantation versus perfusion-assisted methods, as well as the spectrum of underlying tumors. Additionally, survival outcomes are inconsistently reported, with quantitative synthesis being limited, thus leaving the overall safety profile and survival patterns inadequately defined [9, 10].

This study systematically reviews the existing literature on IATx, employing meta-analytic methods to quantitatively synthesize key perioperative and oncologic outcomes. Furthermore, it summarizes the reported survival outcomes to better characterize overall survival (OS) following IATx. By integrating current evidence, this study aims to provide a comprehensive evaluation of the safety and oncologic effectiveness of IATx for complex intra-abdominal malignancies.

## Methods

### Study design and reporting standards

This systematic review and single-arm meta-analysis was conducted in accordance with the Preferred Reporting Items for Systematic Reviews and Meta-Analyses (PRISMA) 2020 guidelines. The review protocol was registered with PROSPERO (registration number: CRD420251816; Supplementary Document S1). During the review process, minor amendments were made to the title and analytical details to accurately reflect the available evidence; however, these changes did not affect the original objectives or scope of the study [11, 12].

### Literature search strategy

A comprehensive literature search was executed across PubMed, Embase, Scopus, Web of Science, and the Cochrane Library, covering each database from inception to December 4, 2025. Medical Subject Headings (MeSH) and free-text terms related to IATx and *ex vivo* resection techniques were utilized. Key search terms included “intestinal autotransplantation,” “*ex vivo* resection,” “intestinal auto-transplantation,” “autograft,” and “*ex vivo* intestinal transplantation.” No restrictions were imposed based on language or publication date. Reference lists of included studies were manually screened for relevant articles, and forward citation tracking was performed where applicable (Supplementary Table S1 in Supplementary Document S2).

### Inclusion criteria

Studies were eligible for inclusion if they met the following criteria: (1) patients underwent IATx; (2) the studies focused on complex intra-abdominal diseases, benign or malignant, deemed unresectable *in situ*; (3) at least one extractable perioperative outcome, pathological result, survival-related variable, or graft-related event was reported; and (4) the study employed any clinical design, including retrospective or prospective cohort studies, institutional experiences, or case series.

The intervention of interest comprised *ex vivo* resection in conjunction with IATx. All variations—full *ex vivo*, partial *ex vivo*, and modified *ex vivo* IATx—were considered eligible. Approved procedures included extracorporeal or partially extracorporeal resections under vascular interruption, followed by vascular reconstruction and reimplantation of the intestinal segment.

### Exclusion criteria

Studies were excluded if they (1) involved animal models, experimental bench studies, or solely perfusion simulations without clinical implantation; (2) were reviews, editorials, commentaries, or conference abstracts lacking extractable clinical data; (3) reported conventional *in situ* resections or vascular reconstructions without IATx; (4) involved donor-derived intestinal transplantation, isolated small-bowel transplantation, or simultaneous autotransplantation of multiple organs, making it impossible to evaluate outcomes specific to IATx separately; or (5) represented duplicate publications or overlapping patient cohorts, in which case the most comprehensive report was retained. Single-patient case reports were excluded unless they provided unique, clinically relevant information directly aligned with the review objectives.

### Data extraction

Data were independently extracted by two reviewers using a standardized Microsoft Excel spreadsheet. The extracted variables included (1) study characteristics (first author, year of publication, country, and study design); (2) patient and disease characteristics (sample size, age, sex, disease etiology, and follow-up duration); and (3) surgical and clinical outcomes, encompassing operative details, perioperative parameters, postoperative complications, pathological outcomes, and reported survival information. Any discrepancies were resolved through discussion.

### Quality assessment

The methodological quality of the included studies was evaluated using the Methodological Index for Non-Randomized Studies (MINORS) [13]. Since all eligible studies were non-comparative, the eight-item MINORS checklist was utilized, with each item scored from 0 to 2, yielding a maximum score of 16. Quality assessment was independently performed by two reviewers, with any disagreements resolved through consensus. Item-level MINORS scores and justifications for each included study are presented in Supplementary  Table S2 in Supplementary Document S2.

### Statistical analysis

Quantitative synthesis was conducted using single-arm random-effects meta-analysis (DerSimonian–Laird method). Pooled 7estimates were calculated with random-effects models to accommodate anticipated clinical and methodological heterogeneity across studies. In instances where multiple publications reported overlapping cohorts from the same institution and time period, all relevant reports were included in the qualitative synthesis; however, only one publication was incorporated into the meta-analysis to prevent double-counting. The study with the most comprehensive extractable data for the outcomes of interest was chosen for quantitative synthesis.

### Perioperative outcomes

For continuous outcomes, such as cold ischemia time (CIT), operative time, and postoperative length of stay, pooled means were computed. When studies provided medians and ranges (or interquartile ranges), means and standard deviations were estimated using the approximation methods by Hozo *et al.* [14] and Wan *et al.* [15], with the formula standard deviation (SD) ≈ (max–min)/4 applied for sample sizes (*n*) ≤ 70. For proportion-based outcomes, including 90-day mortality, major complications, and R0 resection rates, variance-stabilizing transformations (Freeman-Tukey double arcsine) were applied prior to pooling.


$$\text{SD}\approx \frac{\text{Max-Min}}{4}(N\leq 70)$$

### Survival outcomes

Survival outcomes were pooled separately for each reported time point, with the 3-year OS as the primary endpoint, to avoid extrapolation beyond available data. For studies providing individual patient survival data, Kaplan–Meier survival probabilities and 95% confidence intervals (CIs) were derived using Greenwood’s formula. For studies that reported only time-specific survival rates and sample sizes, but did not provide individual-level data or exact CIs, approximate 95% CIs were estimated by using the Wilson score method for binomial proportions. Study-level survival estimates were pooled by using random-effects meta-analysis after log(−log) transformation.

### Heterogeneity and sensitivity analyses

Heterogeneity was assessed using the Cochran *Q* test and the *I*^2^ statistic, where *I*^2^ > 50% indicated substantial heterogeneity. Leave-one-out sensitivity analyses were conducted for outcomes exhibiting moderate-to-high heterogeneity to evaluate robustness. Additional sensitivity checks included the exclusion of studies with extreme estimates and the application of alternative transformation methods. All analyses were performed in R (version 4.3.2; R Foundation for Statistical Computing, Vienna, Austria) utilizing the meta and survival packages.

## Results

### Study selection

A total of 30,010 records were identified via database searches. Following the removal of 14,533 duplicates, 15,477 records underwent title and abstract screening, resulting in the exclusion of 14,677 records. Full texts were requested for 800 articles, of which 752 were excluded based on assessments that highlighted non-autologous models, esophageal reconstruction, living-donor or allergenic intestinal transplantation, reviews, or other non-original clinical studies.

**Figure 1:**
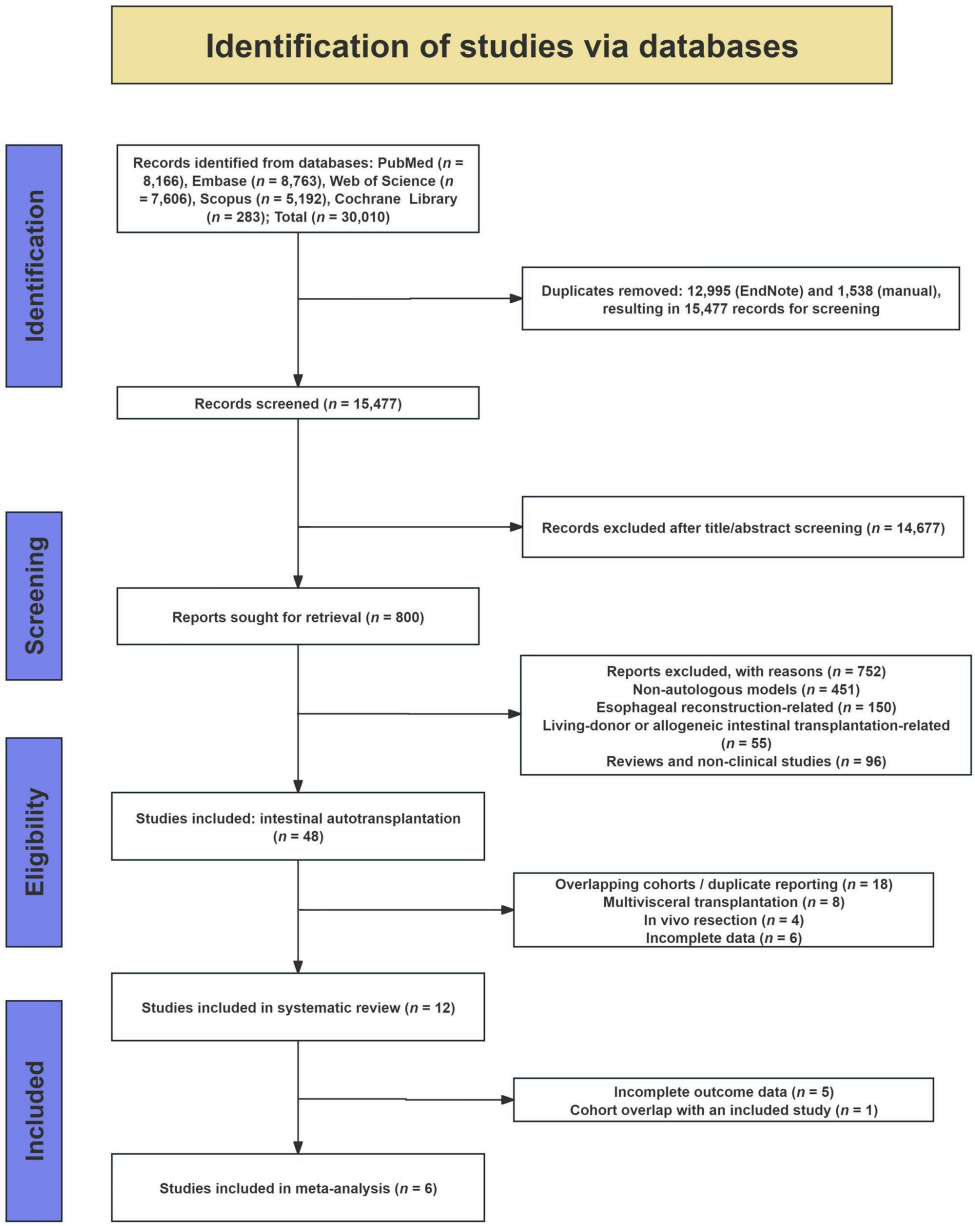
PRISMA flow diagram of study selection process. Records were identified through database searches (PubMed, Embase, Web of Science, Scopus, and the Cochrane Library), duplicates were removed, and titles/abstracts were screened. Full-text reports were assessed for eligibility and excluded, with reasons provided for each. A total of 12 studies were included in the systematic review, and 6 in the meta-analysis. Exclusions at the eligibility stage included overlapping cohorts/duplicate reporting, multivisceral transplantation, *in vivo* resection, and incomplete data; additional exclusions from meta-analysis were due to incomplete outcome data or cohort overlap with an included study.

Ultimately, 48 full-text articles were evaluated for eligibility, leading to the exclusion of 36 studies due to overlapping cohorts/duplicate reporting, multivisceral transplantation, *in vivo* resection, or incomplete data. This process resulted in the inclusion of 12 studies in the systematic review. Out of these, six studies provided extractable outcome data and were incorporated into the quantitative meta-analysis; the remaining six were excluded from pooling due to non-extractable outcomes (*n *= 5) or cohort overlap with an included study (*n *= 1). In situations where multiple publications from the same institution reported overlapping cohorts, the most comprehensive dataset was kept for quantitative analysis to avoid double-counting (Fig. 1).

### Study characteristics

The final selection included 12 studies: four case reports, one case series, four retrospective cohort studies, and one multicenter retrospective study; additionally, two conference abstracts with extractable outcome data were identified and included where applicable. The majority of studies originated from China (9/12), primarily from the First Affiliated Hospital of Zhejiang University in Hangzhou, China. A multicenter study was conducted across the United States, Italy, and Germany (including Columbia University and the University of Miami), while one study was from Iran.

Overall, these studies reported on 160 patients, representing the entirety of published global clinical experience to date. IATx was the most frequently reported surgical approach. Indications for IATx were heterogeneous and included pancreatic malignancies (pancreatic ductal adenocarcinoma and locally advanced pancreatic cancer), colorectal cancer, sarcomas, and various complex mesenteric root lesions (Table 1). Six studies, contributing data from 122 patients, were included in the quantitative meta-analysis (Table 2).

**Table 1:** Characteristics of studies included in the systematic review and meta-analysis of intestinal autotransplantation for complex mesenteric root tumors

Study	Authors, Year, reference	Country	Institution	Study design	Sample size (*n*)	Age, median (range)	Study period	Tumor type	Procedure type	Included in systematic review	Included in meta-analysis	Reason not pooled
1	Li *et al.*, 2025 [21]	China	Sichuan Provincial People’s Hospital	Case series	19	61 (56–65)	June 2022 to January 2024	PDAC = 14 ASCP = 1 LPS = 1 CCA = 1 MF = 2	*In vivo* hypothermic perfusion + IATx	Yes	Yes	
2	Liang *et al.*, 2023 [5]	China	Zhejiang University First Affiliated Hospital	Retrospective cohort	36	60 (39–71)	August 2019 to November 2022	PDAC = 36	*Ex vivo* + IATx	Yes	Yes	
3	Wu *et al.*, 2025 [7]	China	Zhejiang University First Affiliated Hospital	Retrospective cohort	10	55 (32–71)	May 2018 to December 2022	LACC = 2 LRCC = 8	*Ex vivo* + IATx	Yes	Yes	
4	Qiao *et al.*, 2024 [1]	China	Zhejiang University First Affiliated Hospital	Retrospective cohort	48	60.6 (43–76)	August 1, 2019 to January 31, 2024	LAPC = 48	*Ex vivo* + IATx	Yes	No	Overlapping cohort with Study 2 (duplicate population)
5	Wu *et al.*, 2019 [3]	China	Xijing Hospital, Fourth Military Medical University	Retrospective cohort	15	44.9 (20–67)	January 2011 to January 2018	PDAC = 3 AC = 2 SPN = 1 NET = 1 LMS = 1 SCN = 1 MF = 4 GN = 1 PA = 1	*Ex vivo* + IATx	Yes	Yes	
6	Zeng *et al.*, 2008 [25]	China	West China Hospital, Sichuan University	Case	1	21	2007	Enormous cavernous hemangioma of the small intestine mesentery	*Ex vivo* + IATx	Yes	No	No extractable data for the outcome
7	Wei *et al.*, 2019 [26]	China	Xijing Hospital, Fourth Military Medical University	Case	1	54	2019	Superior mesenteric artery dissection	*Ex vivo* + IATx	Yes	No	No extractable data for the outcome
8	Liu *et al.*, 2023 [27]	China	PLA Rocket Force Characteristic Medical Center	Case	2	66, 58	2023	LAPC = 2	*Ex vivo* + IATx	Yes	No	No extractable data for the outcome
9	Cheng *et al.*, 2018 [28]	China	Union Hospital, Huazhong University of Science and Technology	Case	1	34	2017	Large mesenteric desmoid tumor secondary to familial adenomatous polyposis	*Ex vivo* + IATx	Yes	No	No extractable data for the outcome
10	Fujiwara *et al.*, 2025 [2]	USA, Italy, Germany	(1) Columbia University, USA; (2) University of Miami, USA; (3) Asklepios Hospital Barmbek, Semmelweis University Campus, Germany; (4) University of Bologna; (5) University of Illinois, USA	Retrospective cohort	35	31 (15–51)	1999–2024	PDAC = 7 PSC = 1 mCCA = 1 SBAC = 2 LPS = 2 LMS = 2 ES = 1 MF = 9 NET = 2 SPT = 3 IMT = 1 IPMN-IS = 1 LYM = 1 HEM-E = 1 HEM-M = 1	*Ex vivo*+IATx	Yes	Yes	
11	Nikeghbalian *et al.*, 2011 [23]	IRAN	Shiraz Transplant Research Center	Meeting abstract	7	NA	August 2010 to April 2011	LAPC = 6 RMS = 1	*Ex vivo*+IATx	Yes	Yes	
12	Nogoud *et al.*, 2019 [24]	IRAN, SUDAN	Multiple Institutions (Sudan, Iran)	Meeting abstract	21	NA	January 2015 to June 2016	NA	*Ex vivo*+IATx	Yes	No	No extractable data for the outcome

Notes: (1) Abbreviations: PDAC = pancreatic ductal adenocarcinoma, ASPC = pancreatic adenosquamous carcinoma, LPS = liposarcoma, CCA = cholangiocarcinoma, MF = mesenteric fibromatosis, IATx = intestinal autotransplantation, GN = ganglioneuroma, PA = pseudoaneurysm, SCN = serous cystic neoplasm, NET = neuroendocrine tumor, AC = adenocarcinoma (duodenum/colon), SPN/SPT = solid pseudopapillary neoplasm/tumor, PSC = pancreatic squamous carcinoma, SBAC = small bowel adenocarcinoma, mCCA = metastatic cholangiocarcinoma, ES = Ewing sarcoma, IMT = inflammatory myofibroblastic tumor, IPMN-IS = intraductal papillary mucinous neoplasm with *in situ* carcinoma, LYM = lymphoma, HEM-E = hemangioendothelioma, HEM-M = hemangiomatosis, RMS = rhabdomyosarcoma, GIST = gastrointestinal stromal tumor, LAPC = locally advanced pancreatic cancer, LACC = locally advanced colon cancer, LRCC = locally recurrent colon cancer.

(2) Duplicate cohort: Study 4 includes an overlapping patient cohort with the Zhejiang University study published in 2023 (Study 2). Therefore, Study 4 was included in the systematic review only and excluded from the meta-analysis to avoid double counting; Study 2 was included in the meta-analysis.

**Table 2:** Perioperative and oncologic outcomes of intestinal autotransplantation for complex mesenteric root tumors (studies included in the meta-analysis)

Authors, Year, Reference	F/M	Neoadjuvant therapy, % (*n*/*N*)	R0 (%)	Operation time (h)	WIT, minutes	CIT, minutes	Perfusion type	**Severe complications** (≥**Grade III), *n*/*N***	Perioperative mortality (90 days), *n*/*N*	Recurrence & metastasis, *n*/*N*	Postoperative hospital stay, median (range), days	Alive at last follow-up, Alive/total, *n*/*N*	Follow-up, median (range), months	Malignant/benign tumor proportion	OS (%)
Li *et al.*, 2025, [21]	9/10	82.35% (14/17)	100%	10.5 ± 3.3	3.7 ± 2.2	52.2 ± 17.6	*In vivo*	1/19	0	6/19	NR	18/19	21.3 ± 5.3	17/19 (89.5%)	1-year OS = 100% 2-year OS = 93.3%
Wu *et al.*, 2019, [3]	6/9	55.56% (5/9)	100%	10.7 ± 3.42	2.4 (1.8–3.5）	198.8 (135–250)	*Ex vivo*	8/15	1/15	4/19	21（10–68）	12/15	29.9（3.1–89.6）	9/15 (60%)	1-year OS = 85.70% 2-year OS = 71.40 % 3-year OS = 46.30 %
Liang *et al.*, 2023, [5]	18/18	100% (36/36)	94.4%	8.98 ± 2.31	0.733（0.05–2.517	106.5 (40–270)	*Ex vivo*	16/36	3/36	16/36	34（14–154）	19/36	14.5 (5.7–20.9)	100% malignant tumor	1-year OS = 87.47% 2-year OS = 42.08 % 3-yer OS = 31.56%
Wu *et al.*, 2025, [7]	2/8	40% (4/10)	100%	8.37 ± 1.22	36 (25–51)	110 (44–185)	*Ex vivo*	5/10	0	2/10	21（10–36）	8/10	23.9 (10.1–92.0)	100% malignant tumor	1-year OS = 100% 2-year OS = 100% 3-year OS = 80%
Fujiwara*et al.*, 2025, [2]	13/22	37.1% (13/35)	85.7%	12.5 ± 3.75	31 (15–51)	154 (74–250)	*Ex vivo*	4/35	5	NR	23 (10–82)	NR	55 (range NR)	16/19	1-year OS = 87.7% 3-year OS = 70.30% 5-year OS = 66.2%
Nikeghbaliane *et al.*, 2011, [23]	NR	NR	100%	11.5 ± 1.2	NR	NR	*Ex vivo*	1/7	1	NR	NR	2/7	NR	100% malignant tumor	NR

Note: F/M = female/male, WIT = warm ischemia time, CIT = cold ischemia time, OS = overall survival, NR = not reported.

Given the complexity of IATx and potential variations in patient selection among institutions, specific inclusion and exclusion criteria for each study were detailed and summarized (Table 3). The majority of the studies predominantly enrolled patients with locally advanced malignancies involving major mesenteric vessels, for whom standard resections could not achieve an R0 margin.

**Table 3:** Summary of inclusion criteria and surgical details across included meta studies

Authors, Year, Reference	Tumor types and cohort	Surgical procedures	Detailed surgical indications and selection criteria	Core anatomical and biological commonality
Li *et al.*, 2025, [21]	Total *n* = 19 PDAC: 14 (locally advanced) ASCP: 1 LPS: 1 CCA: 1 MF: 2 (mesenteric fibromatosis)	*In vivo* hypothermic perfusion + IATx combined with pancreatoduodenectomy and resection of invaded mesenteric root vessels.	**Anatomical criteria:** (1) Tumors invading SMA and SMV; (2) SMA invasion > 180; (3) Length of small intestine with an intact marginal artery distal to the affected artery ≥ 1 meter. **Biological/Oncological criteria:** (1) Optimal physical condition; (2) Significantly low or stabilized CA19-9 levels following neoadjuvant therapy; (3) Tumor at stable/locally progressed stage without distant metastasis.	(1) Extensive involvement of the mesenteric root vascular system (SMA, SMV); (2) Conventional radical resection cannot achieve R0 status while preserving sufficient functional small bowel. This necessitates the temporary division and re-establishment of small bowel blood supply to perform complex tumor resection safely.
Liang *et al.*, 2023, [5]	Total *n* = 36 LAPC	*Ex vivo* IATx combined with various pancreatic resections (Whipple, Total or Distal Panc,reatectomy) Involves complete resection and reconstruction of SMA and/or CA	**Anatomical criteria:** (1) SMA invasion length > 4 cm; (2) Tumor contacting SMA root > 180°, precluding direct end-to-end anastomosis; (3) Distal SMA invasion around J1A bifurcation (>180°), conventionally unreconstructible; (4) Long-segment PV/SMV invasion (>5 cm) involving major venous confluences; (5) CA or CHA involvement. **Biological/Oncological Criteria**:(1) Favorable response to neoadjuvant FOLFIRINOX ± anti-PD-1; （2） Young age, good performance status, dramatic CA19-9 decrease, and significant tumor shrinkage.	(1) Locally advanced PDAC extensively invading major mesenteric vessels (SMA/CA, PV/SMV). (2) This extreme invasion pattern makes conventional R0 resection impossible or severely jeopardizes bowel perfusion. IATx serves as the ultimate salvage technique to protect/re-establish vascularization, aiming to maximize R0 resection rates.
Wu *et al.*, 2025, [7]	Total *n* = 10 LACC: 2 LRCC: 8	*Ex vivo* IATx combined with pancreaticoduodenectomy (*n* = 8) or IATx alone (*n* = 2). Includes en bloc SMA resection	**Anatomical criteria**: (1) Tumor contact with the SMA > 180; (2) Absence of locoregional peritoneal, pelvic, or distant metastasis. **Biological/Oncological criteria**:(1) Failed standard systemic chemotherapy or showed no benefit; (2) For recurrent disease (LRCC), a disease-free interval of ≥ 6 months; (3) Favorable evaluation by a multidisciplinary team (MDT)	(1) Locally advanced or recurrent colon cancer extensively invading the SMA root; (2) Similar to pancreatic indications, this degree of arterial invasion represents a technical contraindication to conventional surgery. IATx facilitates en bloc SMA resection to achieve R0 margins while preventing irreversible intestinal ischemia
Wu *et al.*, 2019, [3]	Total *n* = 15 Heterogeneous neoplasms including: PDAC: 3 Desmoid tumor: 4 Colon/Duodenal adenocarcinoma: 2 Neuroendocrine: 1 Sarcoma/Neuroma: 2 Pseudoaneurysm: 1 Others: 2	*Ex vivo* IATx combined with pancreaticoduodenectomy (*n* = 13) or local resection (*n* = 2)	**Anatomical criteria:** (1) Locally invasive neoplasms arising from the pancreas, duodenum, mesentery, or retroperitoneum with involvement of the SMA. **Biological/Oncological criteria:** (1) No evidence of distant metastases (except for highly selected neuroendocrine tumors with resectable liver metastasis); (2) Exclusion of poorly differentiated neoplasms or PDAC locally encasing the celiac trunk; (3) Evaluated by MDT.	**(1) Tumors of various origins sharing identical anatomical constraints (extensive SMA involvement);** (2) Standard surgical techniques for these patients would result in uncontrolled bleeding, irreversible bowel ischemia, or non-curative (R1/R2) resection. IATx expands the eligibility for curative-intent surgery
Fujiwara *et al.*, 2025, [2]	Total *n* = 35 Highly malignant (*n* = 16): PDAC, sarcomas, small bowel adenocarcinoma, etc. Benign/Low-grade (*n* = 19): Desmoid tumors, NETs, SPN, etc.	*Ex vivo* IATx (ERIA) with Whipple procedure (*n* = 25) or without (*n* = 10). Involves back-table vascular reconstruction	**Anatomical criteria:** (1) Tumors encasing the mesenteric root, potentially involving up to second-order vessels. (2) Declined for surgery elsewhere; deemed completely unresectable by conventional in situ methods. **Biological/Oncological criteria:** (1) Preserved functional status; (2) For malignant: ≥ 6 months of tumor stability or response on chemotherapy; no extra-regional lymphadenopathy; (3) For benign/low-grade: Severe complications (obstruction, PV thrombosis, severe pain); evaluated for allotransplant as backup.	(1) Massive mesenteric root vascular encasement across highly diverse histology; (2) Regardless of the tumor’s biological aggressiveness, all patients faced an identical technical predicament: conventional resection would cause massive bowel loss. IATx provided the only technical pathway to R0/complete resection.

PDAC = pancreatic ductal adenocarcinoma, ASPC = pancreatic adenosquamous carcinoma, LPS = liposarcoma, CCA = cholangiocarcinoma, MF = mesenteric fibromatosis, LAPC = locally advanced pancreatic cancer, LACC = locally advanced colon cancer, LRCC = locally recurrent colon cancer, NET = neuroendocrine tumor, SPN = solid pseudopapillary neoplasm, IATx = intestinal autotransplantation, SMA = superior mesenteric artery, SMV = superior mesenteric vein, PV = portal vein, SMV = superior mesenteric vein, CA = celiac artery, CHA = common hepatic artery.

### Quality assessment

Methodological quality of the included non-comparative studies was assessed using the MINORS. Since most studies were single-arm retrospective case series without control groups, only the first eight MINORS items were applied (maximum score: 16), with each item scored as 0 (not reported), 1 (reported but inadequate), or 2 (reported and adequate). Two reviewers independently conducted the assessment, with any disagreements resolved by consensus. Conference abstracts were not evaluated using MINORS due to limited methodological reporting; however, when extractable outcome data were available, they were included in the meta-analysis. Sensitivity analyses excluding conference abstracts were performed to evaluate the robustness of the pooled estimates (Supplementary  Table S2 in Supplementary Document S2).

## Results of meta-analysis

### Overall survival

**Figure 2:**
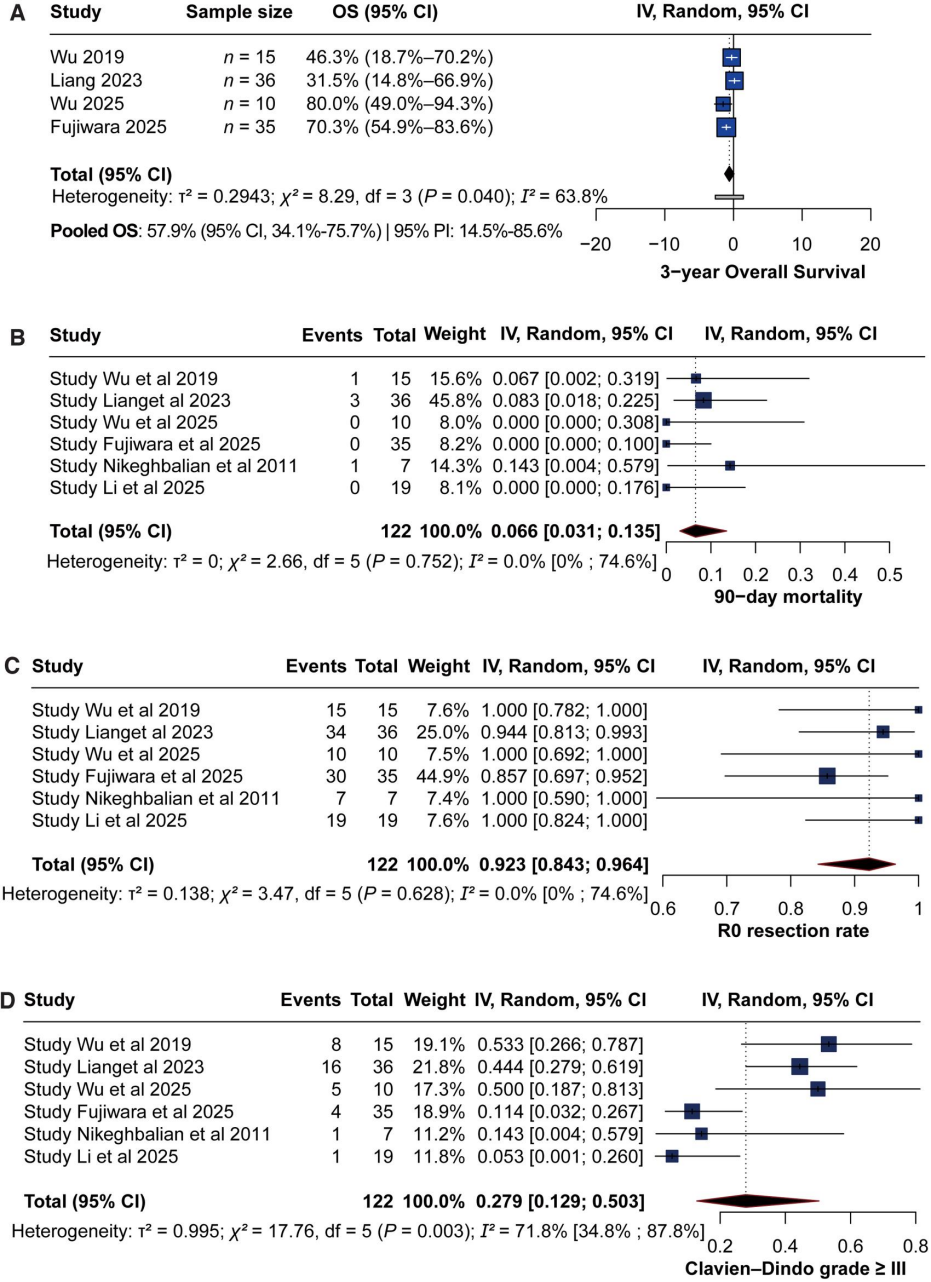
Forest plots of the primary outcomes. (A) Pooled 3-year OS. (B) Pooled 90-day postoperative mortality. (C) Pooled R0 resection rate. (D) Pooled rate of severe postoperative complications (Clavien–Dindo grade ≥ III). Pooled estimates were calculated using a random-effects model, with individual study proportions shown as squares (size proportional to study weight) and 95% CIs as horizontal lines; the pooled effect is shown as a diamond. Heterogeneity was assessed by using *I*^2^ and *χ*^2^ statistics. OS = overall survival, CI = confidence interval.

Four studies provided extractable data on the 3-year OS, defined as the primary survival endpoint. The pooled 3-year OS rate was found to be 57.9% (95% CI, 34.1%–75.7%). Moderate and statistically significant between-study heterogeneity was observed (*I*^2^ = 63.8%, *P *= 0.040), resulting in a relatively wide 95% prediction interval ranging from 14.5% to 85.6%, indicating considerable variability in mid-term survival outcomes across different clinical settings (Fig. 2A). One-year OS data were reported in five studies; due to substantial between-study heterogeneity (*I*^2^ = 85.3%, *P *< 0.001), this pooled estimate is presented in Supplementary  Fig. S1 in Supplementary Document S2.

### Perioperative safety outcomes

Six studies (122 patients) reported on 90-day mortality, yielding a pooled rate of 6.6% (95% CI, 3.1%–13.5%; *I*^2^ = 0%, *P *= 0.752) (Fig. 2B). Major postoperative complications, classified as Clavien–Dindo grade ≥ III, were documented in the same six studies, with a pooled incidence of 27.9% (95% CI, 12.9%–50.3%; *I*^2^ = 72%, *P *= 0.003) (Fig. 2D).

### R0 resection rate

R0 resection rates were reported in six studies (122 patients), resulting in a pooled rate of 92.3% (95% CI, 84.3%–96.4%; *I*^2^ = 0%, *P *= 0.628). This high R0 rate likely reflects the technical advantages of *ex vivo* resection and IATx, enabling complete en bloc tumor removal with extensive vascular and mesenteric clearance under controlled conditions (Fig. 2C).

### Perioperative outcomes

**Figure 3:**
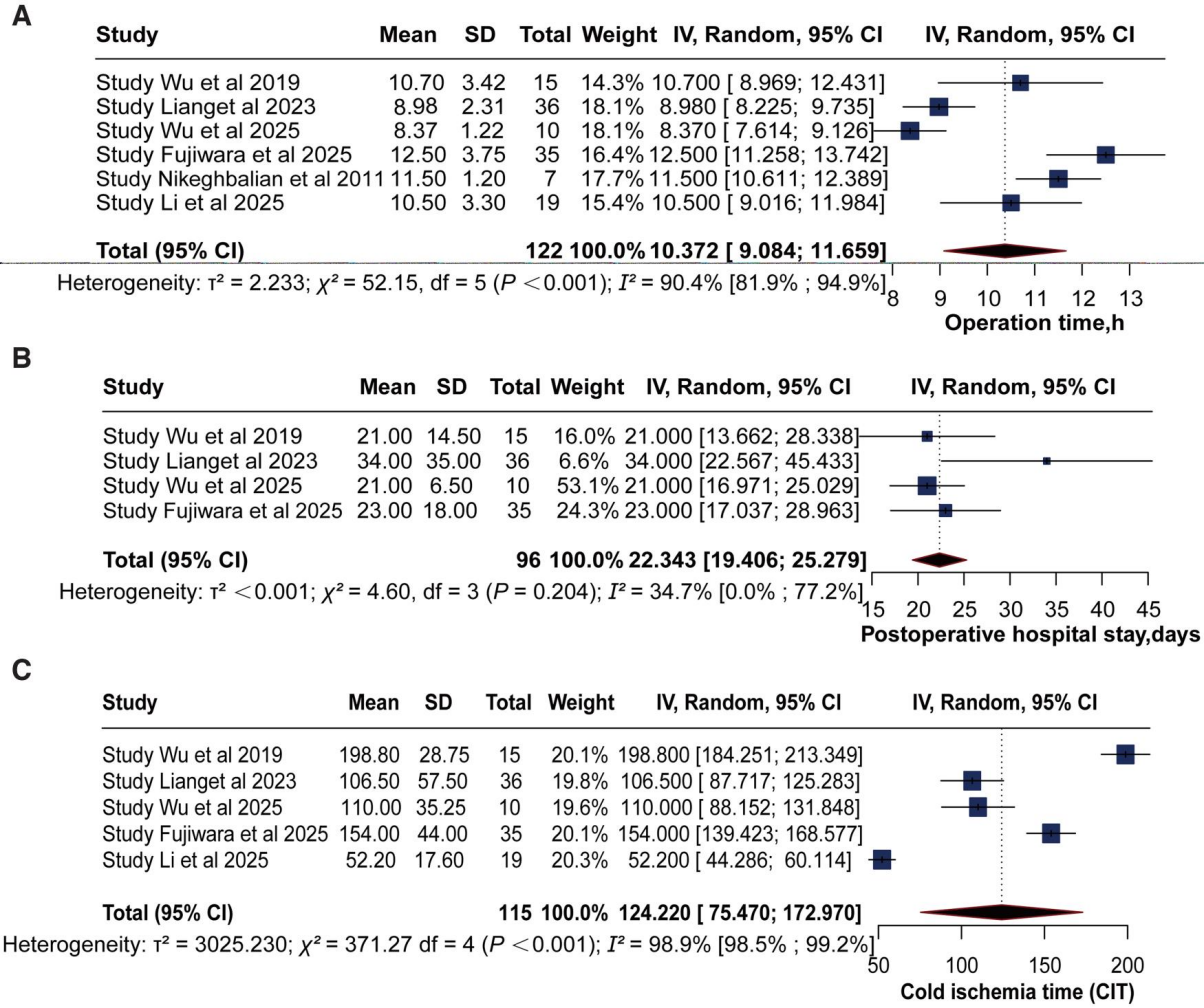
Forest plots of secondary perioperative outcomes. (A) Operation time (h). (B) Postoperative hospital stay (days). (C) CIT. Pooled mean estimates were calculated using a random-effects model. Individual study means are shown as squares (size proportional to study weight) with 95% CIs indicated by horizontal lines; pooled estimates are shown as diamonds. Between-study heterogeneity was assessed using the *I*^2^ and *χ*^2^ statistics. CIT = cold ischemia time, CI = confidence interval.

Perioperative outcomes were analyzed using random-effects models. The CIT, reported in five studies (115 patients), had a pooled mean of 124 minutes (95% CI, 75–173 minutes; *I*^2^ = 98.9%, *P *< 0.001). The operative time, available from six studies (122 patients), was reported as 10.4 hours (95% CI, 9.1–11.7 hours; *I*^2^ = 90.4%, *P *< 0.001). The postoperative length of hospital stay reported in five studies (96 patients) averaged 22 days (95% CI, 19–25 days; *I*^2^ = 35%, *P *= 0.203). The substantial heterogeneity in CIT and operative time likely reflects differences in surgical techniques, the extent of vascular reconstruction, and institutional learning curves (Fig. 3).

### Sensitivity analysis

**Figure 4:**
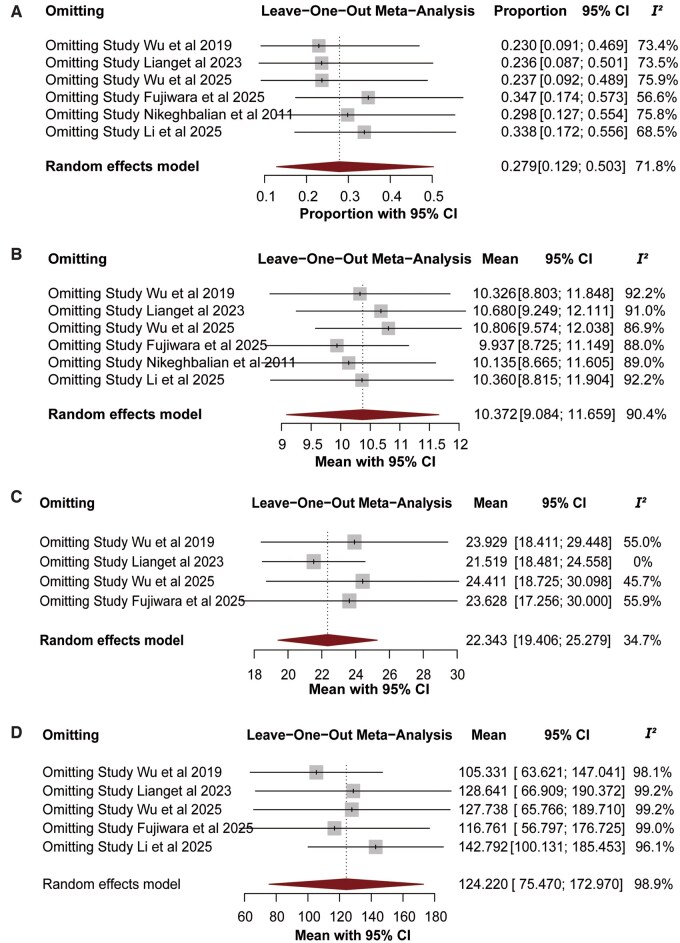
Leave-one-out sensitivity analyses for pooled estimates. (A) Rate of severe postoperative complications (Clavien–Dindo grade ≥ III). (B) Operation time (h). (C) Postoperative hospital stay (days). (D) CIT. Each row shows the pooled estimate recalculated after omitting one study at a time (random-effects model). Squares indicate the leave-one-out pooled estimates with 95% CIs, and the diamond represents the overall pooled estimate using all included studies; *I*^2^ values are reported for heterogeneity. CIT = cold ischemia time, CI = confidence interval.

Leave-one-out sensitivity analyses were performed to assess the robustness of the primary findings for outcomes showing moderate-to-high heterogeneity. For major postoperative complications (Clavien–Dindo grade ≥ III), the pooled incidence remained stable across iterations, ranging from 23.0% to 34.7%, with heterogeneity (*I*^2^ = 71.8%) varying from 56.6% to 75.9%, indicating that no single study materially influenced the pooled estimate. Similarly, for CIT and operative time, pooled mean estimates remained stable (CIT: 105–143 minutes; operative time: 9.9–10.8 hours), while heterogeneity persisted in all scenarios (CIT: *I*^2^ > 95%; operative time: *I*^2^ > 87%). Given the observational nature of the evidence base and procedural heterogeneity across centers, these results should be interpreted as reflecting clinical and methodological variability rather than the influence of any single study. Overall, the pooled results were robust against the exclusion of individual studies, with no single study disproportionately affecting the summary estimates (Fig. 4).

### Subgroup analysis of perioperative outcomes

**Figure 5:**
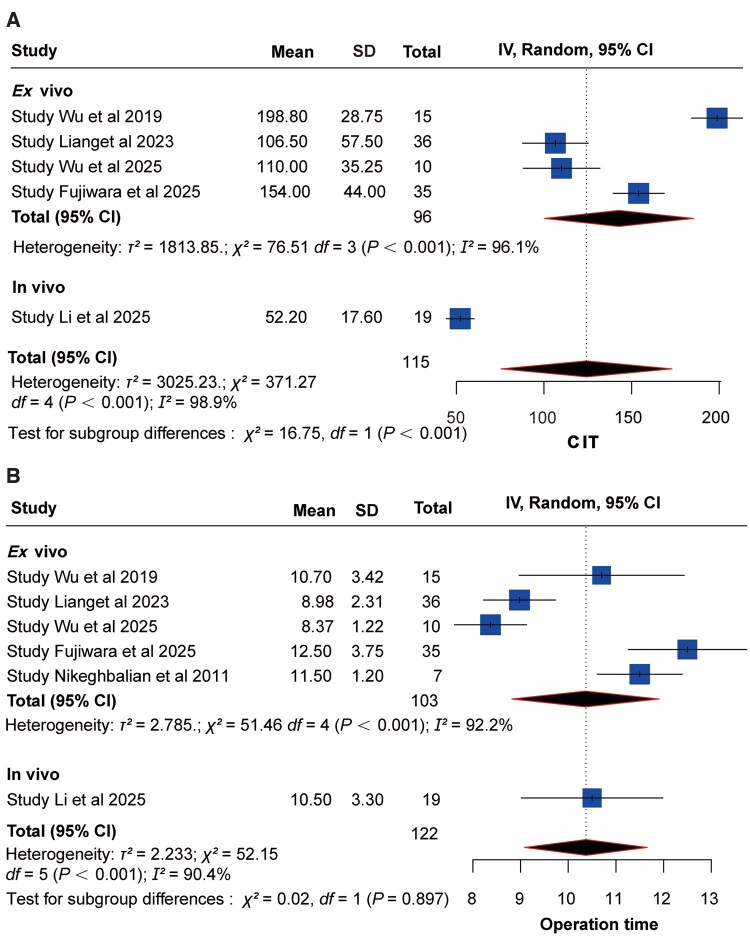
Subgroup analyses of perioperative time parameters stratified by surgical perfusion technique (*ex vivo* vs *in vivo*). (A) Forest plot comparing CIT between the *ex vivo* and *in vivo* approaches, demonstrating a statistically significant difference between the subgroups (*P *< 0.001). (B) Forest plot comparing total operation time between the two approaches, showing no significant difference (*P *= 0.898). Individual study mean values are presented with horizontal lines indicating the 95% CIs. The size of each square is proportional to the study’s statistical weight in the meta-analysis. The pooled mean estimates for each subgroup and the overall cohort are also indicated. Random-effects models were applied for both analyses. SD, standard deviation; CIT = cold ischemia time, CI = confidence interval, IV = inverse variance.

To investigate the sources of the extreme statistical heterogeneity (*I*^2^ > 90%) noted in perioperative parameters, subgroup analyses were conducted based on the surgical perfusion technique (*ex vivo* vs. *in vivo*). For CIT (Fig. 5A), the analysis indicated a statistically significant difference between the two techniques (*P *< 0.001). As expected, the *in vivo* cohort displayed a considerably shorter CIT (mean: 52.20 minutes) compared to the *ex vivo* cohort. This substantial between-group variance suggests that the choice of surgical technique is a key determinant of CIT. Nonetheless, severe statistical heterogeneity persisted within the *ex vivo* subgroup (*I*^2^ = 96.1%).

In contrast, the subgroup analysis for overall operative time did not reveal a significant difference between the two techniques (*P *= 0.898) (Fig. 5B). The *in vivo* study reported an operative time of 10.50 hours, which was closely aligned with the pooled estimate for the *ex vivo* group. Consistent with the findings for CIT, the internal heterogeneity within the *ex vivo* cohort remained notably high (*I*^2^ = 92.2%).

## Discussion

IATx combined with *ex vivo* resection represents a highly specialized and technically challenging approach aimed at achieving R0 resection in patients with locally advanced intra-abdominal malignancies that involve critical mesenteric vessels—diseases often deemed unresectable with conventional *in situ* en bloc techniques. This systematic review and single-arm meta-analysis synthesized all published clinical experiences to date (12 studies involving 160 patients), offering the most comprehensive summary of perioperative safety and mid-term oncologic outcomes available for this technique. The pooled 3-year OS rate was 57.9% (95% CI, 34.1%–75.7%), while the R0 resection rate achieved was 92.3% (95% CI, 84.3%–96.4%). Additionally, the 90-day mortality rate was 6.6% (95% CI, 3.1%–13.5%), and the incidence of major complications (Clavien–Dindo grade ≥ III) was reported at 27.9% (95% CI, 12.9%–50.3%). Collectively, these findings suggest that IATx with *ex vivo* resection may provide a high likelihood of margin-negative resections and encouraging mid-term survival in carefully selected patients treated at experienced centers, albeit with significant perioperative morbidity.

Notably, the disease spectrum and clinical context of patients included in this meta-analysis differ from those reported in many studies on conventional combined resections; thus, direct cross-cohort comparisons should be interpreted with caution and should not be construed as demonstrating superiority [16]. IATx is predominantly utilized for aggressive conditions such as pancreatic head tumors or mesenteric root sarcomas with major vascular involvement—cases that have historically been classified as “unresectable” and linked to poor prognoses (for instance, the 5-year OS rate for locally advanced pancreatic cancer typically falls below 10% under non-surgical management) [17]. For contextual comparison, locally advanced colon cancer (LACC) invading the duodenum and pancreas can achieve 5-year survival rates of 49%–66.3% when managed with pancreatoduodenectomy combined with right hemicolectomy after R0 resection [18]. Similarly, in cases of locoregionally recurrent colon cancer, R0 resection has been associated with approximate 3- and 5-year survival rates of 58% and 52%, respectively, whereas outcomes after R1/R2 resections substantially decline (with 3-year survival rates of 27% and down to 11%, respectively) [19, 20]. Against this background—and recognizing the limitations of comparability—our pooled 3-year OS rate of 57.9% alongside an R0 resection rate of 92.3% suggests that *ex vivo* IATx could present a viable pathway to achieving margin-negative resections and favorable mid-term outcomes for selected patients with mesenteric root involvement, particularly when conventional *in situ* en bloc resection is not technically feasible. However, the extent of any relative benefit remains uncertain and necessitates further validation in more comparable cohorts or prospective studies.

These considerations are particularly pertinent for locally advanced pancreatic ductal adenocarcinoma with major vascular encasement, which has traditionally been classified as “unresectable” [21]. Thus, the morbidity associated with IATx should not be directly compared to standard pancreaticoduodenectomy for resectable diseases, as the patient populations differ significantly. A more appropriate clinical benchmark is pancreatectomy with concurrent arterial resection (AR) for LAPC post-neoadjuvant therapy. A recent meta-analysis by Xue *et al.* [22] focusing on this specific cohort reported a pooled morbidity of 51% and a perioperative mortality rate of 2%. Compared to this comparable baseline, the perioperative risk profile in our IATx cohort—major complications of approximately 27.9% and 90-day mortality of 6.6%—appears commensurate with the inherent risks associated with extensive vascular reconstruction in locally advanced disease.

Importantly, while conventional *in situ* AR achieved a pooled R0 resection rate of 79% and a median 3-year OS rate of 51.6% in the same analysis, the IATx cohort demonstrated an R0 rate of 92.3% and a pooled 3-year OS rate of 65.3%. This comparison suggests that in cases where conventional *in situ* approaches face anatomical constraints and challenges in margin assessment, the *ex vivo* IATx technique could effectively overcome these technical barriers. Thus, IATx represents a feasible pathway to margin-negative resection with curative intent for a select patient population that would otherwise face palliative care or be considered non-resectable [23, 24].

The high pooled R0 resection rate may be attributed to several intrinsic advantages of the *ex vivo* approach, including a bloodless operative field, improved exposure for en bloc resection, the capability for extensive mesenteric and vascular dissection, and intraoperative frozen-section assessment for margin control. However, it is essential to recognize that the available evidence primarily stems from a limited number of high-volume centers (predominantly in China), and various sources of bias must be acknowledged. Patient selection is likely stringent, and variations in institutional practices concerning perioperative management and pathological assessment may exist.

This study observed substantial heterogeneity in operative parameters (*I*^2^ > 90% for CIT and operative time), likely reflecting differences in surgical strategies (complete vs. partial *ex vivo* resection), the extent of reconstruction, center experience, and case complexity. In contrast, heterogeneity was moderate for 3-year OS (*I*^2^ = 63.8%) and minimal for R0 resection rate and 90-day mortality, suggesting that within experienced programs, margin status and early postoperative mortality may remain relatively consistent, while survival outcomes are more sensitive to tumor type, systemic therapy, and follow-up practices. Future studies should therefore aim to report outcomes with clinically relevant stratifications based on tumor histology, patterns of vascular involvement, neoadjuvant treatment approaches, and the scope of reconstruction to enhance interpretability and generalizability.

This study presents significant limitations. First, all included studies were retrospective, single-arm series characterized by low-to-moderate methodological quality (median MINORS score approximately 10/16) and restricted external validity. Second, some survival estimates relied on aggregated data; in cases where precise Kaplan–Meier intervals were unavailable, CIs were approximated using Wilson binomial methods, which may introduce imprecision. Furthermore, long-term outcomes beyond 5 years, recurrence patterns, quality of life, nutritional status, and graft bowel function were infrequently reported, hindering a thorough assessment of durable benefits and functional trade-offs.

Despite these limitations, findings indicate that *ex vivo* resection combined with IATx may represent a potentially curative option for a narrowly defined patient population with mesenteric root involvement, who are otherwise unlikely to achieve an R0 resection through conventional techniques, especially when neoadjuvant strategies do not render the disease resectable. Given its complexity, this approach should be performed in advanced multidisciplinary centers with expertise in pancreatic surgery, vascular reconstruction, and transplant surgery, supported by comprehensive perioperative critical care. Future efforts should focus on establishing prospective international registries, standardized technical and reporting frameworks, and multicenter observational studies. Conducting comparative effectiveness research with well-matched contemporaneous cohorts, where feasible, will be essential to elucidate the role of IATx within current treatment pathways.

In conclusion, *ex vivo* resection combined with IATx provides promising mid-term survival and an excellent R0 resection rate for carefully selected patients with complex locally advanced intra-abdominal malignancies, albeit with substantial perioperative morbidity. Broader implementation will necessitate higher-quality evidence and extended follow-up to determine optimal patient selection and long-term outcomes.

## Supplementary Material

goag074_Supplementary_Data

## Data Availability

The dataset for this article is publicly available, available upon reasonable request, or data sharing.
