# Supplementary material for: Intestinal autotransplantation for complex intra-abdominal diseases previously considered unresectable: a systematic review and single-arm meta-analysis
Source: Gastroenterol Rep (Oxf). 2026 Jul 17;14:goag074. doi: 10.1093/gastro/goag074 (PMC13378460; doi:10.1093/gastro/goag074)
Supplement: goag074_Supplementary_Data [file goag074_supplementary_data.docx]

**Supplementary Table S1. Detailed search strategies and yields across databases (last search: 4 December 2025)**

| **Database** | **Date of search (repeated search)** | **Search query** | **Number of found publications (repeated search)** |
| --- | --- | --- | --- |
| PubMed | **4 December 2025** | (("small intestine" OR "small bowel" OR intestine OR jejunum OR ileum OR "Intestine, Small"[Mesh] OR "Jejunum"[Mesh] OR "Ileum"[Mesh] OR "Intestine, Small/transplantation"[Mesh]) AND ("Transplantation, Autologous"[Mesh] OR "Autografts"[Mesh] OR "Replantation"[Mesh] OR autotransplant*[tiab] OR autograft*[tiab] OR "autologous transplant*"[tiab] OR "auto-transplant*"[tiab] OR "autogenous transplant*"[tiab] OR "intestinal autotransplantation"[tiab] OR "bowel autotransplantation"[tiab] OR ex vivo OR ex-vivo OR ex situ OR ex-situ OR extracorpor*)) | 8,166 |
| Cochrance | **4 December 2025** | #1 MeSH descriptor: [Intestine, Small] explode all trees  #2 ("small intestine" OR "small bowel" OR intestine OR bowel OR jejunum OR ileum OR intestinal)  #3 MeSH descriptor: [Transplantation, Autologous] explode all trees  #4 (autotransplant* OR autograft* OR ("autologous NEXT transplant") OR ("auto NEXT transplant") OR ("autogenous NEXT transplant")  OR "intestinal autotransplantation" OR "bowel autotransplantation")  #5 (ex vivo OR ex-vivo OR ex situ OR ex-situ OR extracorpor*)  #6 (#1 OR #2) AND (#3 OR #4 OR #5) | 283 |
| Embase | **4 December 2025** | ('small intestine'/exp OR 'small bowel' OR intestine OR jejunum OR ileum OR 'jejunum'/exp OR 'ileum'/exp) AND ('autotransplantation'/exp OR 'autograft'/exp OR autotransplant* OR autograft* OR 'autologous transplant*' OR 'auto-transplant*' OR 'autogenous transplant*' OR 'intestinal autotransplantation' OR 'bowel autotransplantation' OR 'ex vivo' OR 'ex situ') | 8,763 |
| Scopus | **4 December 2025** | TITLE-ABS-KEY ( "small intestine" OR "small bowel" OR intestine OR jejunum OR ileum ) AND TITLE-ABS-KEY ( autotransplant* OR autograft* OR extracorpor* OR "autologous transplant" OR "auto transplant" OR "auto-transplantation" OR "autogenous transplant" OR "intestinal autotransplantation" OR "bowel autotransplantation" OR "ex vivo" OR "ex situ" ) AND ( LIMIT-TO ( SUBJAREA , "MEDI" ) ) | 5,192 |
| Web of Science | **4 December 2025** | ("intestine" OR "small intestine" OR small bowel OR jejunum OR ileum OR bowel OR intestinal)AND("autologous transplantation" OR autologous transplant* OR autotransplant* OR autograft* OR "auto-transplant*" OR "autogenous transplant*" OR "intestinal autotransplantation" OR "bowel autotransplantation" OR ex vivo OR ex-vivo OR ex situ OR ex-situ OR extracorpor*) | 7,606 |

**Supplementary Table S2. MINORS assessment (item-level scores and justifications) for studies included in the meta-analysis.**

| **Study**  **(First author, year)** | **Country** | **n** | **Stated aim** | **Consecutive patients** | **Prospective data** | **Appropriate endpoints** | **Unbiased assessment** | **Appropriate follow-up** | **Loss to follow-up ≤5%** | **Sample size calculation** | **Total** | **Quality** |
| --- | --- | --- | --- | --- | --- | --- | --- | --- | --- | --- | --- | --- |
| Li et al., 2025 | China | 19 | 2 | 2 | 0 | 2 | 1 | 2 | 2 | 0 | 11 | Moderate |
| Wu et al.，2019 | China | 15 | 2 | 2 | 0 | 2 | 1 | 2 | 2 | 0 | 11 | Moderate |
| Wu et al.，2025 | China | 10 | 2 | 2 | 0 | 2 | 1 | 2 | 2 | 0 | 11 | Moderate |
| Liang et al，2023 | China | 36 | 2 | 2 | 1 | 2 | 1 | 2 | 2 | 0 | 11 | Moderate |
| Fujiwara et al. (2025) | USA | 35 | 2 | 2 | 0 | 2 | 1 | 2 | 2 | 0 | 11 | Moderate |

The Methodological Index for Non-Randomized Studies (MINORS) was used to assess the methodological quality of the included non-comparative studies.

Since all included studies were single-arm retrospective case series without a control group, only the first 8 items of the MINORS tool were applied (maximum score 16 points).Each item was scored as follows:0 = not reported,1 = reported but inadequate,2 = reported and adequate.

The 8 items were as follows : (1)a clearly stated aim; (2) inclusion of consecutive patients ; (3) prospective collection of data ; (4) endpoints appropriate to the aim of the study ; (5) unbiased assessment of the study endpoint ; (6) follow-up period appropriate to the aim of the study ; (7) loss to follow-up not exceeding 5% ;and (8) prospective calculation of the study size.

Quality classification:(1)13–16 points = high quality (2)9–12 points = moderate quality (3)≤8 points = low quality

Two reviewers independently performed the assessment, with discrepancies resolved by consensus.

**1. Article: Li et al. (2025) – *In vivo* hypothermic perfusion intestinal auto-transplantation for patients with tumors invading the mesenteric root vascular system: an unusual consecutive 19-case series
Total score: 11/16 (Moderate quality)**

| Item | MINORS Criterion | Score (0–2) | Justification |
| --- | --- | --- | --- |
| 1 | A clearly stated aim | 2 | Clearly stated: to investigate safety, feasibility, and outcomes of in vivo hypothermic perfusion combined with IATx for mesenteric root tumors invading vascular system. |
| 2 | Inclusion of consecutive patients | 2 | Explicitly described as “consecutive 19 patients” between June 2022 and January 2024 at a single center. |
| 3 | Prospective collection of data | 0 | Retrospective study |
| 4 | Endpoints appropriate to the aim of the study | 2 | Endpoints (R0 resection rate, operative time, ischemia times, complications [Clavien-Dindo], recurrence, metastasis, survival, follow-up) fully appropriate to safety and oncological outcomes. |
| 5 | Unbiased assessment of the study endpoint | 1 | Complications graded by standard Clavien-Dindo classification; pathological margins and vascular invasion confirmed by independent pathology; blood supply assessed objectively with indocyanine green fluorescence and ultrasound. No evidence of bias. |
| 6 | Follow-up period appropriate to the aim of the study | 2 | Median follow-up 23 months (mean 21.3 ± 5.3 months, latest follow-up February 2025), adequate for assessing perioperative complications, early recurrence, and short-term oncological outcomes in this aggressive tumor setting. |
| 7 | Loss to follow-up less than 5% | 2 | No patients lost to follow-up reported; all 19 patients followed via outpatient visits and telephone interviews. |
| 8 | Prospective calculation of the study size | 0 | No sample size calculation or power analysis reported |

**2.Wu G et al. (2019) – Perioperative complications and outcomes after intestinal autotransplantation for neoplasms involving the superior mesenteric artery (*n* = 15 patients)**

| Item | MINORS Criterion | Score (0–2) | Justification |
| --- | --- | --- | --- |
| 1 | A clearly stated aim | 2 | Clearly stated aim: to describe surgical indications, postoperative complications, and clinical outcomes after IATx for neoplasms involving the SMA. |
| 2 | Inclusion of consecutive patients | 2 | Explicitly described as 15 consecutive patients undergoing IATx from January 2011 to January 2018. |
| 3 | Prospective collection of data | 0 | Retrospective analysis using data retrieved from prospectively maintained databases and medical records; no prospective data collection protocol. |
| 4 | Endpoints appropriate to the aim of the study | 2 | Endpoints (perioperative complications, mortality, R0 resection, recurrence, survival, nutritional outcomes) fully appropriate to the study aim. |
| 5 | Unbiased assessment of the study endpoint | 1 | Complications classified using standard definitions (e.g., ISGPS for pancreatic fistula, DGE, hemorrhage); pathology-confirmed margins and vascular invasion; vascular patency assessed objectively with ultrasound and CT angiography; no evident bias. |
| 6 | Follow-up period appropriate to the aim of the study | 2 | Median follow-up 29.9 months (range 3.1–89.6 months); adequate for assessing perioperative complications, recurrence, and medium-term oncological and nutritional outcomes in this aggressive tumor setting. |
| 7 | Loss to follow-up less than 5% | 2 | All 15 patients followed; no loss to follow-up reported (follow-up described for all surviving patients). |
| 8 | Prospective calculation of the study size | 0 | No prospective sample size or power calculation reported |

**3.Wu G et al. (2025) – Intestinal autotransplantation for locally advanced or locally recurrent colon cancer invading superior mesenteric artery (*n* = 10 patients）**

| Item | MINORS Criterion | Score (0–2) | Justification |
| --- | --- | --- | --- |
| 1 | A clearly stated aim | 2 | Clearly stated aim: to examine the safety, feasibility, and outcomes of IATx in patients with locally advanced or recurrent colon cancer invading the SMA. |
| 2 | Inclusion of consecutive patients | 2 | Described as a retrospective analysis of all eligible patients undergoing IATx between May 2018 and December 2022 at two centers; inclusion/exclusion criteria explicitly stated, implying consecutive eligible cases. |
| 3 | Prospective collection of data | 0 | Explicitly stated as a “retrospective cohort study” with “retrospective analysis of prospectively collected data”; no prospective data collection protocol for the study itself. |
| 4 | Endpoints appropriate to the aim of the study | 2 | Endpoints (R0 resection rate, perioperative complications graded by Clavien-Dindo, recurrence-free survival, overall survival, nutritional outcomes) fully appropriate to the aim of assessing safety and efficacy. |
| 5 | Unbiased assessment of the study endpoint | 1 | Margins confirmed by pathology; complications objectively graded using Clavien-Dindo classification; vascular patency assessed with Doppler ultrasound/CT angiography; recurrence detected by routine CT; no evident bias in reporting. |
| 6 | Follow-up period appropriate to the aim of the study | 2 | Mean follow-up 23.9 months (with Kaplan-Meier estimates at 3 years); sufficient for mid-term oncological outcomes (recurrence-free and overall survival) in locally advanced/recurrent colon cancer. |
| 7 | Loss to follow-up less than 5% | 2 | All 10 patients accounted for in follow-up; no loss to follow-up reported. |
| 8 | Prospective calculation of the study size | 0 | No prospective sample size or power calculation reported (typical for case series). |

### 4.Scoring Table for "Radical resection combined with intestinal autotransplantation for locally advanced pancreatic cancer after neoadjuvant therapy: a report of 36 consecutive cases"

| **Item No** | **MINORS Item (English)** | **Score**  **(0-2)** | **Detailed comments (based on content)** |
| --- | --- | --- | --- |
| 1 | A clearly stated aim | 2 | The aim is clearly stated: "achieve radical resection of locally advanced pancreatic ductal adenocarcinoma (PDAC)..." |
| 2 | Inclusion of consecutive patients | 2 | A total of 36 consecutive cases were included. "Between August 2019 and November 2022, 36 consecutive LAPC patients..." |
| 3 | Prospective collection of data | 1 | Data were retrospectively collected ("Demographic... data... were retrospectively collected"), but the study was registered at clinicaltrial.gov (NCT03983057), with some data from prospective registries. |
| 4 | Endpoints appropriate to the aim of the study | 2 | Endpoints were appropriate, including Clavien-Dindo grade ≥ 3 complications, ICU length of stay, etc. |
| 5 | Unbiased assessment of the study end points | 1 | Tools were used for assessment, but there may be bias in the collection of endpoints. |
| 6 | Follow-up period appropriate to the aim of the study | 2 | Follow-up was at least 15 months, with a median follow-up of 13.6 months, which is appropriate. |
| 7 | Loss to follow-up less than 5% | 2 | Less than 5% loss to follow-up, noted as 0%. |
| 8 | Prospective calculation of the sample size | 1 | No formal statistical sample size calculation was performed, but the study cites itself as the "largest cohort worldwide." |

**5.Article: Fujiwara et al. (2025) – Ex vivo resection and intestinal autotransplantation: an international multicenter study (35 patients)
Total score: 13/16 (High quality)**

| **Item** | **MINORS Item** | **Score**  **(0-2)** | **Detailed Explanation** |
| --- | --- | --- | --- |
| 1 | A clearly stated aim | 2 | The aim is clearly stated in the Abstract and Introduction: “To evaluate the perioperative and long-term outcomes of ex vivo resection and intestinal auto-transplantation (ERIA) for tumors.” The objective is specific, focusing on safety and oncological outcomes in the largest series to date. |
| 2 | Inclusion of consecutive patients | 2 | All cases from five institutions over a defined period (1999–2024) are included; described as a comprehensive multicenter retrospective collection of ERIA cases, implying consecutive enrollment at each center. |
| 3 | Prospective collection of data | 0 | Explicitly described as a “multicenter, international retrospective data analysis”; no prospective design or registry mentioned. |
| 4 | Endpoints appropriate to the aim of the study | 2 | Comprehensive endpoints including operative parameters (time, ischemia times, reconstruction methods), R0 rate (85.7%), perioperative mortality (0%), complications (e.g., graft failure, diarrhea, TPN dependency), hospital stay, readmissions, and long-term OS/RFS (Kaplan-Meier curves stratified by tumor aggressiveness)—fully aligned with perioperative and oncological aims. |
| 5 | Unbiased assessment of the study end points | 1 | Outcomes assessed using objective measures (pathology for margins, imaging/clinical follow-up for recurrence, standard definitions for complications); no blinding or independent adjudication mentioned—partially unbiased, consistent with prior scoring. |
| 6 | Follow-up period appropriate to the aim of the study | 2 | Median follow-up 55.0 months (long enough to report meaningful 1-, 3-, and 5-year OS/RFS with Kaplan-Meier analysis); sufficient for both malignant (highly variable outcomes observed) and benign/low-grade tumors. |
| 7 | Loss to follow-up less than 5% | 2 | All 35 patients included in survival analysis with no mention of loss to follow-up; median follow-up reported for the entire cohort (0% loss implied). |
| 8 | Prospective calculation of the study size | 2 | No formal prospective power calculation (retrospective design), but as the largest multicenter series to date (n=35) with explicit inclusion of all available cases across five high-volume centers, and clear justification as the most comprehensive collection—scored 2 for strong representation of the available population (higher than smaller single-center series). |

**Supplementary Figure S1. One-year OS after IATx**


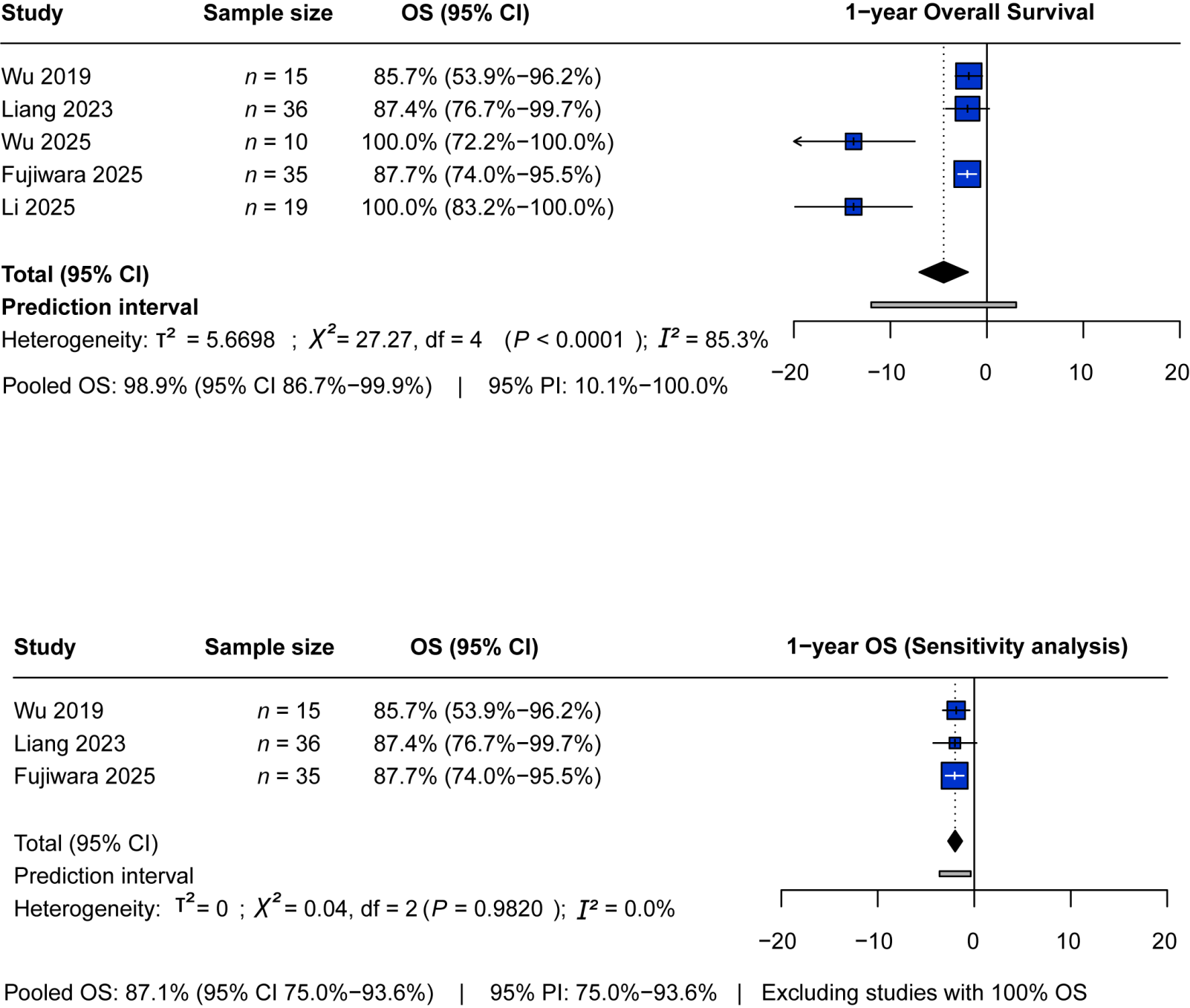


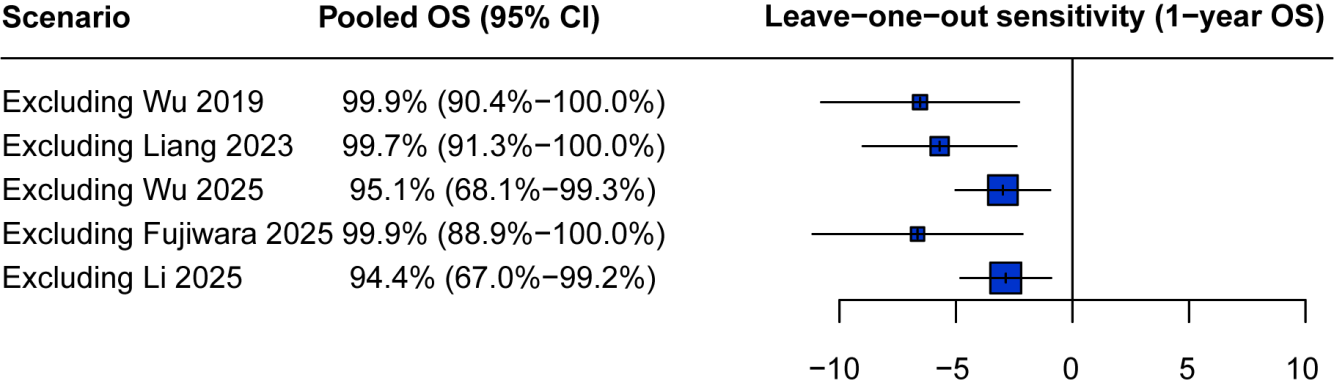


(A) Forest plot of 1-year OS across included studies. (B) Sensitivity analysis excluding studies reporting 100% 1-year OS. (C) Leave-one-out sensitivity analysis for 1-year OS. Pooled estimates were calculated using a random-effects model. Individual study estimates are shown as squares (size proportional to study weight) with 95% confidence intervals (CIs); pooled estimates are shown as diamonds. Between-study heterogeneity was assessed using the Cochran Q test and quantified with I^2^ Prediction intervals (PI) are reported where applicable.OS = overall survival, IATx = intestinal autotransplantation
